# A pooled testing strategy for enterovirus D68 to facilitate local, resource-conserving surveillance

**DOI:** 10.1016/j.diagmicrobio.2025.116934

**Published:** 2025-05-30

**Authors:** Madeline E. Spradley, Timothy Williams, Rendie McHenry, Laura L. Short, Dalton J. Nelson, James D. Chappell, Natasha Halasa, Frederick R. Haselton, Jonathan E. Schmitz

**Affiliations:** a Department of Pathology, Microbiology, and Immunology, Vanderbilt University Medical Center, Nashville, TN, USA; b Current institution: Biodesign Institute, Arizona State University, Tempe, AZ, USA; c Department of Pediatrics, Vanderbilt University Medical Center, Nashville, TN, USA; d Department of Biomedical Engineering, Vanderbilt University, Nashville, TN, USA; e Vanderbilt Institute for Infection, Immunology, and Inflammation, Vanderbilt University Medical Center, Nashville, TN, USA; f Department of Urology, Vanderbilt University Medical Center, Nashville, TN, USA

**Keywords:** Enterovirus D68, Outbreak surveillance, Pooled testing, Respiratory virus, RT-qPCR

## Abstract

Pooled testing represents a resource-conserving diagnostic strategy, and we propose enterovirus-D68 (EV-D68) as an attractive target for pooling (especially locally). To validate an RT-qPCR-based approach, retrospective pools were contrived from EV-D68-positive nasopharyngeal specimens and non-EV-D68-enterovirus-positive specimens. Five/nine-fold pools demonstrated 90.9 %/88.6 % sensitivity versus-unpooled testing (no false-positives), with commensurate results observed prospectively.

## 1. Introduction

Advances in nucleic acid amplification testing (NAAT) have transformed the evaluation of respiratory infections, especially since the emergence of SARS-CoV-2. Diagnostic methods for respiratory viruses now include automated platforms with sample-to-result capabilities; rapid NAATs for point-of-care testing; and highly multiplexed ‘syndromic’ panels [1].

Along with emergent technologies, the pandemic focused attention on long-established strategies to maximize diagnostic throughput/efficiency. In particular, *pooling* is a traditional technique that was applied to SARS-CoV-2 [reviewed in 2], both in resource-abundant [3,4] and resource-limited [5,6] settings. Here, specimens from multiple patients are combined before analysis. When prevalence remains low, most pools test negative, and each patient can be considered negative. Conversely, for positive pools, the individual components can be retested for source-identification.

Pooling can serve diagnostic or epidemiologic roles. In the former, this algorithm (initial pooling, individual repeat-testing) infers a *Detected*/*Not Detected* for each individual specimen. If positivity remains <20 %, optimal pool-sizes can be calculated to maximize resource-conservation [7]. For SARS-CoV-2, several NAATs were FDA-authorized for modest pool-sizes (3–10) that substantially improved workflows [8]. When patient-level diagnoses are not desired, the first step can be used epidemiologically to indicate circulation (including maximum-likelihood estimates of point-prevalence) [9], with further algorithmic modifications possible to account for low prevalence [10].

Simplistically, this process assumes the sensitivity of pooled detection is commensurate to unpooled testing. NAATs are generally well-suited to pooling with low limits-of-detection (LODs). Nevertheless, the concentration of any virus within a specimen follows some distribution [11]. For respiratory viruses, real-time PCR (qPCR) is commonly performed on nasal/nasopharyngeal swabs in transport media, generating C_t_-values. Across patients, some samples demonstrate lower C_t_-values (indicating abundant viral loads), whereas other positive results demonstrate higher C_t_-values. For the latter, dilutional effects from pooling can drive concentrations below assay-LODs, generating false-negative pooled results [12]. This phenomenon, however, must be evaluated empirically for any pathogen, specimen-type, pool-size, and methodology.

COVID-19 now raises an important question: *what other respiratory pathogens could benefit from pooling, diagnostically or epidemiologically*? One possibility is enterovirus D68 (EV-D68), a serotype within the RNA viral genus *Enterovirus*, species D [13]. Although most EV-D68 cases are limited to the upper respiratory tract, severe lung disease can develop, particularly in children and asthma patients [14]. These manifestations are compounded by the uncommon sequela of acute flaccid myelitis, reminiscent of poliovirus myelitis [15]. While EV-D68 circulation is typically low, local upticks in activity are sporadically observed (including with biennial spikes) [16].

EV-D68 testing typically falls outside the purview of individual hospital laboratories. Local capabilities to diagnose enteroviruses (including rhinoviruses) have increased, primarily through syndromic NAATs [17]. Nonetheless, species/serotype-specific testing remains the domain of regional/national public health laboratories; when EV-D68 testing of individual patients is desired, specimens must be referred. Public health and research laboratories are similarly responsible for EV-D68 surveillance [18,19], although with more variable/limited monitoring bandwidth by local clinical labs [20].

This *status quo* can generate challenges for frontline providers. While EV-D68 testing may not be indicated under routine circumstances, needs may change during increased activity. Again, though, information about prevalence is rarely available locally and in real-time. Molecular screening is time/resource-intensive, especially for nonroutine pathogens and unreimbursable testing.

## 2. Methods & results

For EV-D68, even moderate pool-sizes could significantly reduce the effort/cost of surveillance, creating local options that are otherwise impractical. We sought to validate this approach utilizing the most recent RT-qPCR primers/probe-set developed by the CDC for EV-D68 [21,22] (among only a limited number of extant options for this target [23,24]). In light of patient-to-patient C_t_-variability, we first established the diagnostic performance of typical pool-sizes relative to unpooled tests. These efforts (under an IRB protocol) leveraged our institution’s use of the Biofire FilmArray RP2 assay, including its pan-enteroviral result. The assay is employed here for ED/admitted adults and children with compatible symptomatology. Employing a retrospective strategy, we utilized banked enterovirus-positive specimens (nasopharyngeal swabs in universal transport media, frozen after original testing) from July-September 2022, a period of increased EV-D68 activity nationally [20,21].

**Fig. 1.**
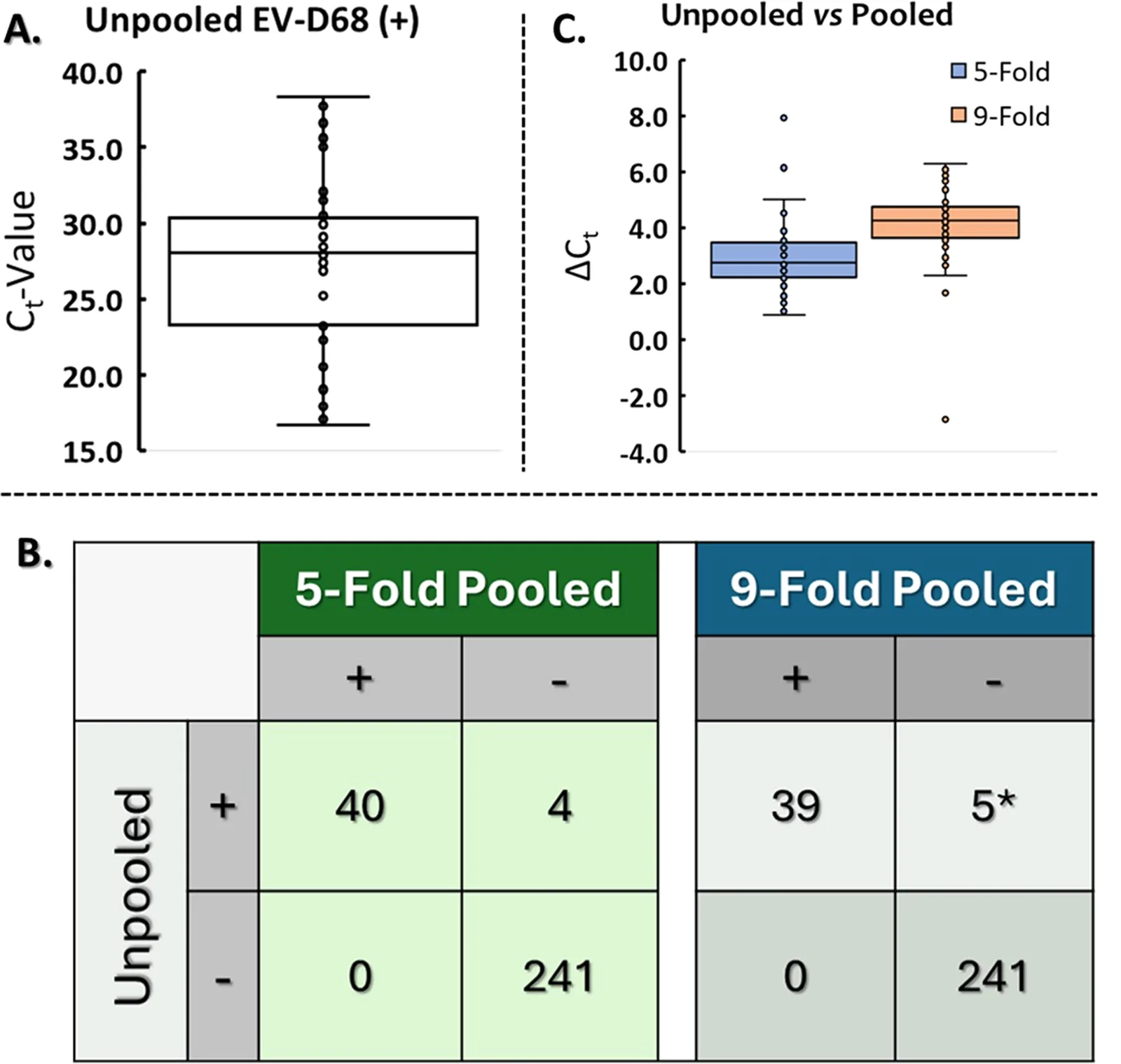
Unpooled versus pooled analysis of EV-D68 positive nasopharyngeal specimens. **[A]** The C_t_-value distribution of 44 EV-D68-positive specimens from our institution, following individual RT-qPCR testing, is summarized here as a box-and-whiskers plot. The median, upper quartile, lower quartile, and total range are all noted. **[B]** Two-by-two contingency tables summarize the agreement between pooled EV-D68 RT-qPCR tests results and the expected results from individual testing, for both 5- and 9-fold pools within a retrospective study. *For 2 (of the 5) false negative specimens in 9-fold pooling, a successful application was observed in one of two technical replicates (although we consider these *categorically* negative for current purposes). **[C]** These box-and-whisker plots depict, for all EV-D68 positive specimens, the change in C_t_ value between individual and pooled testing, again for both 5- and 9-fold pools. These data closely match theoretical predictions for pools of the given sizes. For a single specimen under 9-fold pooling, the pooled result paradoxically demonstrated a lower C_t_ than unpooled specimen; this was an artifact of the high C_t_ value of the unpooled result (nearly 40), outside the assay’s log-linear range.

RNA was extracted from 285 patient-unique enterovirus specimens (18 % adult, 82 % pediatric; median age-3.5 years) via QIAamp Viral RNA-Mini kits; this extraction methodology is well established for locally developed molecular testing for respiratory viruses, and it was part of the first clinical-use protocol authorized by the FDA for SARS-CoV-2 . Extracts were subjected to EV-D68 RT-qPCR via duplicate replicates (QuantStudio-3), yielding 44 EV-D68-*Detected* results (27 % adult, 73 % pediatric; median age-4.5 years) with a C_t_-range of 15.9–40.6 (median-28.5; Fig. 1A). For this study, a specimen was considered positive if both replicates generated detectable C_t_-values. Forty-four unique 5-fold pools and 44 unique 9-fold pools were created with equal volumes of positive specimens, together with enterovirus-positive-EV-D68-negative specimens (from throughout 2022/2023). These specific pool-sizes were motivated by previous norms for pooled viral testing, including the aforementioned SARS-CoV-2 efforts. Two hundred forty-five unique 5-fold pools and 245 unique 9-fold pools were also created with *only* enterovirus-positive-EV-D68-negative specimens. The latter were selected randomly and on an all-comers basis from enterovirus-positive FilmArray specimens, to emulate the process by which real-time, local surveillance would occur.

Coded specimens were transferred to alternate staff for blinded analysis. Relative to unpooled results, 5-fold pools demonstrated 90.9 % positive agreement (40/44; 95-CI: 78.8–96.4 %) and 9-fold pools demonstrated 88.6 % positive agreement (39/44; 95-CI: 76.0–95.5 %) (Fig. 1B). On average, C_t_-values increased by 2.9/4.1 cycles in 5/9-fold pools (Fig. 1C); this observed ΔC_t_ approximates per-cycle amplicon doubling and suggests negligible interference from the pooled matrix background or presence of non-EV-D68 enteroviruses. Under both schemes, the only positive specimens that failed to amplify after pooling demonstrated initial C_t_-values >35. Complete negative agreement was observed for both 5- and 9-fold enterovirus-positive-EV-D68-negative pools (245/245 each; 95-CIs: 98.4–100 %), without any false-positive detections. As to overall agreement (positive and negative), these observations yield Cohen’s kappa values of 0.94 (95-CI: 0.89–0.99) and 0.93 (95-CI: 0.87–0.99) for 5- and 9-fold pools, respectively.

Following this analysis, we next evaluated prospective pooling, commensurate with its application to real-world surveillance. Yet, at any given time, local EV-D68 activity may be low/non-existent, limiting the availability of positive comparators. Therefore, we conducted a *semi-contrived* prospective analysis over 5 weeks (from a period following the conclusion of the above EV-D68 activity-spike). One hundred enterovirus-positive specimens were combined into 5- and 9-fold pools. Pools were formulated in duplicate, and one member of each pair was spiked with EV-D68, emulating low-positive specimens with an unpooled C_t_ ~32. Specimens/pools were analyzed as before, with total concordance of results: individual specimens and unspiked pools were uniformly negative for EV-D68, while spiked pools were all positive.

## 3. Discussion & conclusions

We suggest pooling as a resource-conserving strategy EV-D68 monitoring. Supplementary File 1 presents an analysis of cost savings with the current pooling methodology, as a function of the number enterovirus-positives specimens undergoing testing for EV-D68 (representative of what an institution might encounter routinely). For 20/30/50 weekly specimens, 5-fold pooling reduces (approximate) costs from $249.50/362.50/587.00 to $73.50/98.00/$147.00; 9-fold pooling reduces these figures to $62.50/76.00/103.00. Although the precise savings can depend on local factors, it is clear that even modest pool sizes can significantly reduce the cost of regular EV-D68 surveillance.

With this strategy, we foresee the largest benefit for local epidemiologic surveillance, although a two-step algorithm could also be utilized for patient-level EV-D68 diagnoses (requiring validation in accordance with local regulations for on-site developed diagnostics [25]). Notably, the specimens here were de-identified before analysis, as our intent was individual patient-linkage. While surveillance can inform the need for patient-level testing, it is not a clinical diagnostic in itself. In the case of a two-step pooling algorithm, the pool size could also be varied to maximize resource conservation, depending on positivity estimates. For example, for the 3-month period of EV-positive specimens that we utilized for retrospective validation, the 15 % positivity-rate (44/285) corresponds to 9-fold pooling at optimum [7].

The need for novel EV-D68 monitoring strategies was explored in several recent publications. One group proposed wastewater surveillance to determine if/when local EV-D68 activity is present [26]. In another study, for laboratories that employed FilmArray RP1 testing, the authors demonstrated how PCR melt-curve data (from the component enteroviral reactions) can facilitate EV-D68 surveillance [27]. While not reportable clinically reportable, these raw data are extractable from FilmArray files. Unfortunately, the RP1 assay is discontinued and the RP2 no longer includes these same component-reactions.

The current work represents a further addition to such strategies, relevant across pan-enteroviral testing modalities. The specific dilution schemes evaluated here (5- and 9- fold) were intended to reflect typical pool-sizes for clinical laboratory needs. In theory, pools could be extended further to even larger sizes, although these would have to be validated empirically as further low-C_t_ specimens may evade detection. For instance, based on the observed distribution of unpooled EV-D68 C_t_-values, we anticipate increasing pool-size to 100 would decrease sensitivity to ~80–85 %. At the same time, when discerning an appropriate pool size for local surveillance, a laboratory must consider both theoretical and practical factors. Notably, this includes the total number of samples undergoing surveillance in a given ‘run’. Aside from diminishing marginal savings – see Supplementary File 1 – disproportionately large pooling schemes can limit one’s ability to estimate prevalence rates from a *Detected* result (i.e. if all specimens are consolidated into only 1 or 2 pools). At the same time, more extensive pools could prove valuable if more extensive surveillance were desired by public health authorities in times of low expected EV-D68 prevalence. Alternatively, pooled surveillance could be modified to include *all respiratory specimens* submitted for testing. Here, the size/number of pools would likewise have to increase for commensurate EV-D68 coverage, given inclusion of enterovirus-negative specimens.

Finally, we acknowledge a limitation that this represents a single-institution study, with specimens from one enteroviral season. The C_t_-distribution of EV-D68-positive specimens might differ slightly with broader representation. We also note that the current protocol – with its specific molecular reagents – is not necessarily the only one that might be employed for EV-D68 pooling. There are diverse methodologic variables that an individual laboratory might explore when implementing such testing, or to optimize detection-sensitivity at any given pool size. These could include alternate extraction kits; increasing the volume of extracted (pooled) specimen or additional biochemical (viral precipitation) or biophysical (ultracentrifugation) techniques for selective viral enhancement. Given the high positive agreement of pooled-*vs*-unpooled testing – and, again, the fact that ΔCt-values matched theoretical predictions – we did not attempt further optimization here. However, myriad additional options are potentially available, for which end-users would have to validate their performance.

In summary, we believe the current findings represent a valuable contribution to the field, especially given the challenges of obtaining naturally occurring positive specimens. This study endorses pooled RT-qPCR as a practical solution for EV-D68 monitoring at local levels, generating data that can inform decisions on the need for patient-specific testing.

## Supplementary Material

1

Supplementary material associated with this article can be found, in the online version, at doi:10.1016/j.diagmicrobio.2025.116934.
